# Parasite Co-Infections and Their Impact on Survival of Indigenous Cattle

**DOI:** 10.1371/journal.pone.0076324

**Published:** 2014-02-20

**Authors:** Samuel M. Thumbi, Barend Mark de Clare Bronsvoort, Elizabeth Jane Poole, Henry Kiara, Philip G. Toye, Mary Ndila Mbole-Kariuki, Ilana Conradie, Amy Jennings, Ian Graham Handel, Jacobus Andries Wynand Coetzer, Johan C. A. Steyl, Olivier Hanotte, Mark E. J. Woolhouse

**Affiliations:** 1 Centre for Immunology, Infection and Evolution, University of Edinburgh, Edinburgh, United Kingdom; 2 The Roslin Institute and Royal (Dick) School of Veterinary Studies, University of Edinburgh, Roslin, United Kingdom; 3 International Livestock Research Institute, Nairobi, Kenya; 4 School of Life Science, University of Nottingham, University Park, Nottingham, United Kingdom; 5 Department of Veterinary Tropical Diseases, Faculty of Veterinary Science, University of Pretoria, Onderstepoort, South Africa; 6 Department of Paraclinical Sciences, Faculty of Veterinary Science, University of Pretoria, Onderstepoort, South Africa; Swiss Tropical & Public Health Institute, Switzerland

## Abstract

In natural populations, individuals may be infected with multiple distinct pathogens at a time. These pathogens may act independently or interact with each other and the host through various mechanisms, with resultant varying outcomes on host health and survival. To study effects of pathogens and their interactions on host survival, we followed 548 zebu cattle during their first year of life, determining their infection and clinical status every 5 weeks. Using a combination of clinical signs observed before death, laboratory diagnostic test results, gross-lesions on post-mortem examination, histo-pathology results and survival analysis statistical techniques, cause-specific aetiology for each death case were determined, and effect of co-infections in observed mortality patterns. East Coast fever (ECF) caused by protozoan parasite *Theileria parva* and haemonchosis were the most important diseases associated with calf mortality, together accounting for over half (52%) of all deaths due to infectious diseases. Co-infection with *Trypanosoma* species increased the hazard for ECF death by 6 times (1.4–25; 95% CI). In addition, the hazard for ECF death was increased in the presence of *Strongyle* eggs, and this was burden dependent. An increase by 1000 *Strongyle* eggs per gram of faeces count was associated with a 1.5 times (1.4–1.6; 95% CI) increase in the hazard for ECF mortality. Deaths due to haemonchosis were burden dependent, with a 70% increase in hazard for death for every increase in strongyle eggs per gram count of 1000. These findings have important implications for disease control strategies, suggesting a need to consider co-infections in epidemiological studies as opposed to single-pathogen focus, and benefits of an integrated approach to helminths and East Coast fever disease control.

## Introduction

Natural populations living under wild or field conditions are constantly exposed to a large diversity of parasites. As a result individual hosts, including humans and animals, are frequently co-infected with multiple pathogens either concurrently or in sequence [1]. These multispecies co-infections may result in pathogen-pathogen interactions which may influence the epidemiology of co-infecting parasites [2]–[4] or the consequent effects of infection on host health and performance [5]–[7].

Although co-infections are common in the field and important epidemiologically, most epidemiology studies have focused on single-pathogen infections, and fewer have considered co-infections while assessing the burden of infectious diseases. The last decade has seen increased attention paid to co-infections, with reported studies on animals [3], [7]–[10] and on humans, for example malaria and helminth infections [11] or co-infections involving HIV [12], [13]. From these studies and others, it is evident pathogen-pathogen interactions frequently occur and that their effect will differ both in strength and direction.

Dependent on the mechanisms by which pathogen-pathogen interactions occur, co-infections may cause a) more harm on the host than the combined effect of the component infections, b) less harm than the combined effect of the component infections [14], [15]. The possible mechanisms by which pathogen-pathogen interactions occur modifying host outcomes have been reviewed in detail [2], [16], [17].

Disease-induced mortality will depend on many factors including characteristics of the host, environmental conditions under which the animals are raised, characteristics of infecting pathogens and the pathogen-pathogen interactions in situations where hosts are co-infected. Although most studies on mortality generate useful data on risk factors and mortality rates, the role of co-infections is rarely examined, even in populations where co-infections are known to frequently occur. Knowledge of pathogen-pathogen interactions is limited and we do not know which co-infections are important among domestic animals, and how these influence their survival probabilities. If our understanding on pathogen-pathogen interactions is improved, cost-effective control programs that make use of multispecies approach to the control of morbidity and mortality attributable to infectious diseases may be applied [18], [19]. Our work on mortality of indigenous zebu cattle has identified East Coast fever (ECF), and haemonchosis as the most common definitive aetiological causes of death during the calves’ the first year of life, together accounting for over half (52%) of the observed infectious disease mortalities [20].

This paper investigates the specific risk factors for deaths due to the two main causes of calf mortality in indigenous zebu cattle; ECF and haemonchosis, and tests for the effect size and direction of co-infections on the risk of cause-specific calf mortality. Information on synergistic or antagonistic pathogen-pathogen interactions influencing survival probabilities provide better estimates of the impact of diseases, potentially improving the design of disease control strategies, and ultimately their effectiveness in reducing host mortality.

## Materials and Methods

### Ethics Statement

The study was reviewed and approved by the University of Edinburgh Ethics Committee (reference number OS 03–06), and by the Animal Care and Use Committee (AUCUC) of the International Livestock Research Institute, Nairobi. Standard techniques were used to collect blood and faecal samples for diagnosis and identification of disease and infecting pathogens. The calves were restrained by professional animal health assistants, and by veterinarians. A veterinary surgeon was available to examine any calf falling sick during the course of the study. Any calves in severe distress due to trauma or disease were humanely euthanised by intravenous injection of sodium pentobarbital, administered by a veterinary surgeon. All participating farmers gave informed consent in their native language before recruiting of their animals into the study.

### Study Population

**Figure 1:**
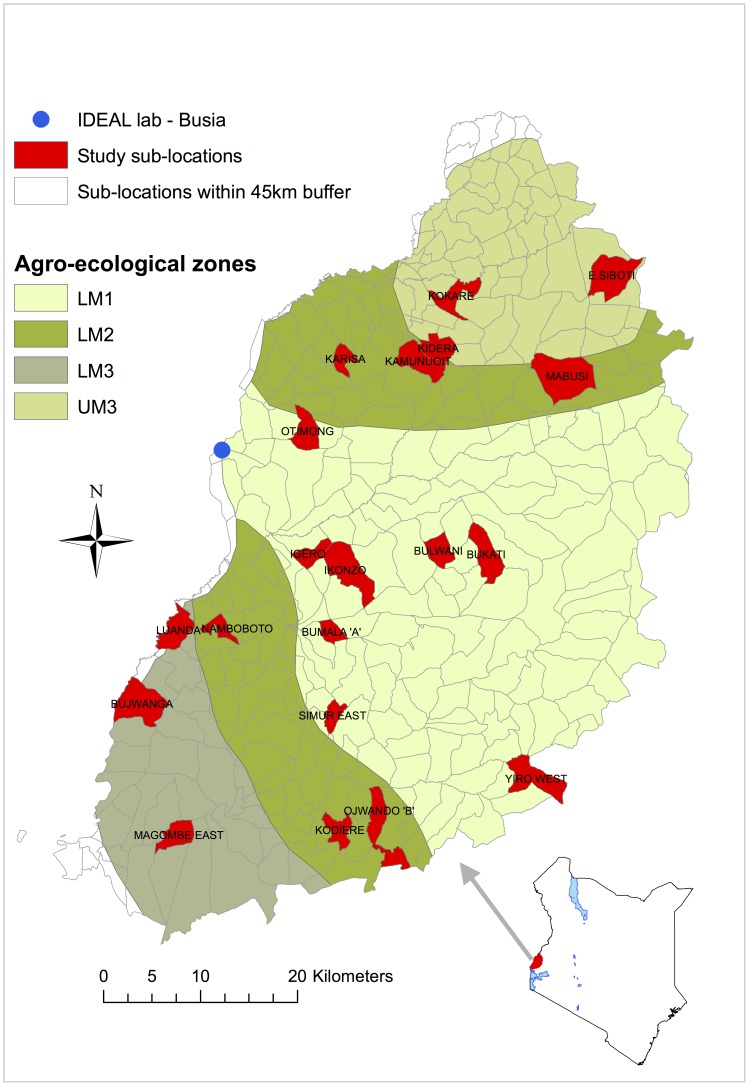
Map of Western Kenya showing the 4 agro-ecological zones and the 20 study sub-locations (in red). The study area comprised sub-locations falling within a 45 km radius from Busia town where the IDEAL project laboratory was located.

The data used in this paper are from the Infectious Disease of East Africa Livestock (IDEAL) cohort study. This study, conducted between October 2007 and September 2010, followed 548 indigenous zebu calves from birth until one year old. The animals came from an area in Western Kenya falling within a 45 km radius of Busia town at the Kenya-Uganda border and covering 4 agro-ecological zones. The study’s field laboratory was located in Busia town, Kenya. Using a stratified (by agro-ecological zone) random cluster sampling approach, study animals were selected from smallholder farms in 20 sub-locations (smallest administrative unit in Kenya). Figure 1 shows the map for the study site. The inclusion criteria required that the calves were recruited into the study within 7 days of birth, be born to a dam that had been on the farm for at least one year, and the calf should have been conceived through natural insemination as opposed to artificial insemination. Additionally, only one calf per farm would be in the study at any one time, and the herd should not be under stall-feeding. The exclusion of herds under stall-feeding and calves from dams artificially inseminated was meant to lower the probability of recruiting crossbred animals. The main production system practiced in the farms was smallholder mixed crop-livestock system. An average farm is 2 hectares in size, grows food crops and keeps approximately 5 cattle among other livestock species. Following recruitment into the study, animals were routinely monitored at 5-week intervals until leaving the study at one year, or until death. The IDEAL cohort study has been described in detail elsewhere, see [21].

### Data Collection

A complete clinical examination was conducted on each study calf at the recruitment visit and during each of the 5-week routine visits. Clinical samples including blood smears, whole blood, serum samples, and faecal samples were collected for screening of pathogens, and measurement of clinical parameters such as total serum proteins and packed cell volume. Live body weight measures (in kilograms) and girth measurements (in centimeters) of the study calves were recorded during the routine visits. Pre-tested questionnaires capturing data on farm characteristics, management practices, herd structure changes, herd health and veterinary treatments were administered at each calf visit. The general body health, udder health, girth measurements and body condition score for each of the study dams were recorded, at each corresponding calf visit. These data on the dam were collected until the study calf was weaned or until leaving the study at one year.

For the study animals that died or that were euthanised during the study, a complete post-mortem (PM) examination was carried out following a standard body system by body system veterinary PM routine [22]. A team of experts reviewed results from parasitological, and histological examination of samples collected at PM, and gross-lesions observed at PM, and determined the specific aetiological cause of death for each case [20].

For the serology data on *Theileria parva, Theileria mutans, Anaplasma marginale* and *Babesia bigemina*, a sero-conversion event was assumed if there was evidence of a rising titre between two consecutive calf visits, and that the titre level was >20 percent positivity (pp).

### Outcome Variable

Two outcome variables were used in this analysis; ECF deaths and haemonchosis deaths. These were defined as deaths in the study animals during the study observation time whose main aetiological cause of death was ECF or haemonchosis respectively. All deaths by cause other than that under investigation were right censored in the analysis.

### Data Analysis

Survival time was defined as the age at which a calf died from the specific aetiological cause under investigation. Cox proportional hazard models as described in Equation 1 were used.



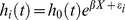
(1)


It expresses the *hazard* at time *t* (i.e the probability of a calf dying from ECF or from haemonchosis at time *t*) as a function of; a) baseline hazard 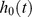 which is the unspecified baseline hazard when the predictors are 0 or absent, b) linear combination of predictors 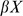 which is an exponential function of a series of variables, and c) cluster term 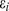 - a random effect accounting for the correlated measurements for study animals from the same study site.

Univariable analysis was carried out with each of the potential non-infectious and infection risk factors listed in Table 1. Factors with a *p* value ≤0.2 were retained and incorporated in the subsequent multivariable model. A backward selection model simplification method was used until only factors significant at a *p* value <0.05 remained in the model. The dropped variables were then added back to the model one at a time to test if there was significant improvement in model fit. The model diagnostics were carried out through graphical evaluation of scaled Schoenfeld residuals plotted against time to test violations of proportional hazards assumption.

**Table 1:** List of covariates tested for their relationship with mortality due to ECF and haemonchosis.

Farm factors	Farmer’s age, gender, education level, main occupation, herd size, land size
Management factors	Tick control, worm control, trypanosome control, vaccine use, grazing practices, watering practices, housing
Maternal status	Heart girth measurement, body condition score, suckling, health condition, dam antibody titres against *Theileria parva, Theileria mutans, Anaplasma marginale, Babesia bigemina*
Environmental variables	Normalised difference vegetation index (NDVI), farm altitude (elevation)
Calf factors	Calf sex, birth weight, heterozygosity, European introgression, clinical episodes, total serum protein, packed cell volume, white blood cell counts
Infectious factors	**ELISA tests (serology):** *Theileria parva, Theileria mutans, Anaplasma marginale, Babesia bigemina,* **Microscopy** *: Trypanosoma spp., Coccidia spp., Theileria spp., Trichophyton* spp., **McMaster microscopy** *: Strongyloides* egg*s, Strongyle* eggs, *Trichuris* spp., *Toxocara vitulorum*, **Sedimentation technique** *: Calicophoron* spp., *Fasciola* spp., **Direct Baermann’s technique** *: Dictyocaulus viviparous,* **Faecal larval cultures** *: Haemonchus placei, Microfilaria* spp., *Oesophagostomum radiatum, Trichostrongylus axei, Cooperia* spp.

For infectious factors, the diagnostic tests (in bold) used are recorded against pathogens identified.

The statistical analysis was done using the *survival* statistical package [23], on the R platform [24]. The raw data used in this study is available from the authors on request.

## Results

A total of 548 calves were recruited and followed up to 51 weeks or until they died, contributing a total of 25,104 calf weeks (481.1 calf years) of life to the study. Five animals were lost to follow due to non-compliance to study protocol or were stolen from the study farms. A total of 88 calves died before reaching 51 weeks of age, giving a crude mortality rate of 16.1 (13.0–19.2; 95% CI) per 100 calves in their first year of life. Of the 88 animals that died, 33 deaths were attributed to East Coast fever, 10 to haemonchosis, and 6 to heartwater. In addition, one death was attributed to each of the following; babesiosis, rabies, salmonellosis, trypanosomiasis, black quarter, viral pneumonia, multifocal abcessation due to *Actinomyces pyogenes*, and *Arcanobacterium* infection. Due to logistical reasons, post mortems were not carried out on 6 of the study calves and the cause of death remained unknown. Seven animals died from known non-infectious causes including trauma, starvation and plant poisoning. The remaining calves were treated as having died from infectious diseases, most with clinical signs indicative of infectious cause but the definitive cause remained unidentified.

**Figure 2:**
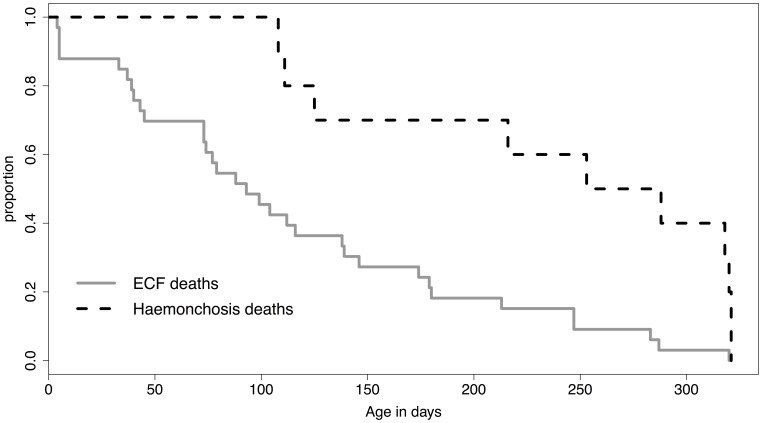
Plot of time to death for ECF and haemonchus deaths, the two main causes of calf mortality causing 33 and 10 deaths respectively. More than 80% of ECF deaths were observed in calves below 6 months of age, whereas most deaths attributed to haemonchosis were in calves older than 6 months.

**Figure 3:**
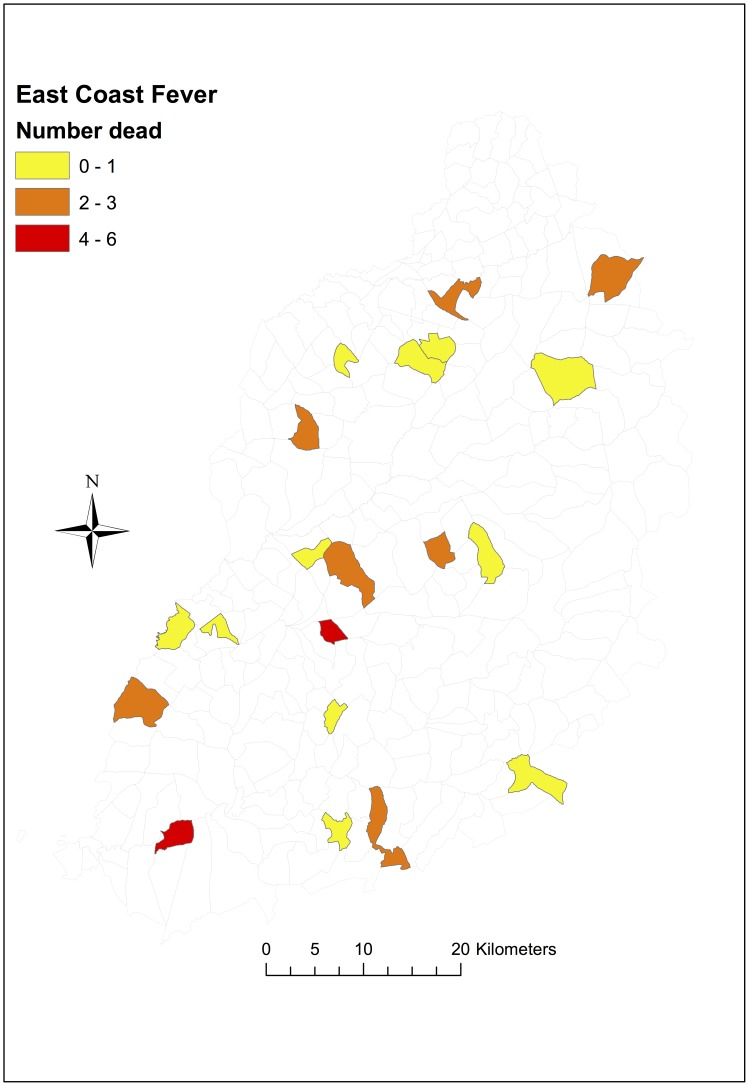
Map showing number of deaths attributable to ECF by sub-location. In total 33 of 88 deaths were attributed to ECF.

**Figure 4:**
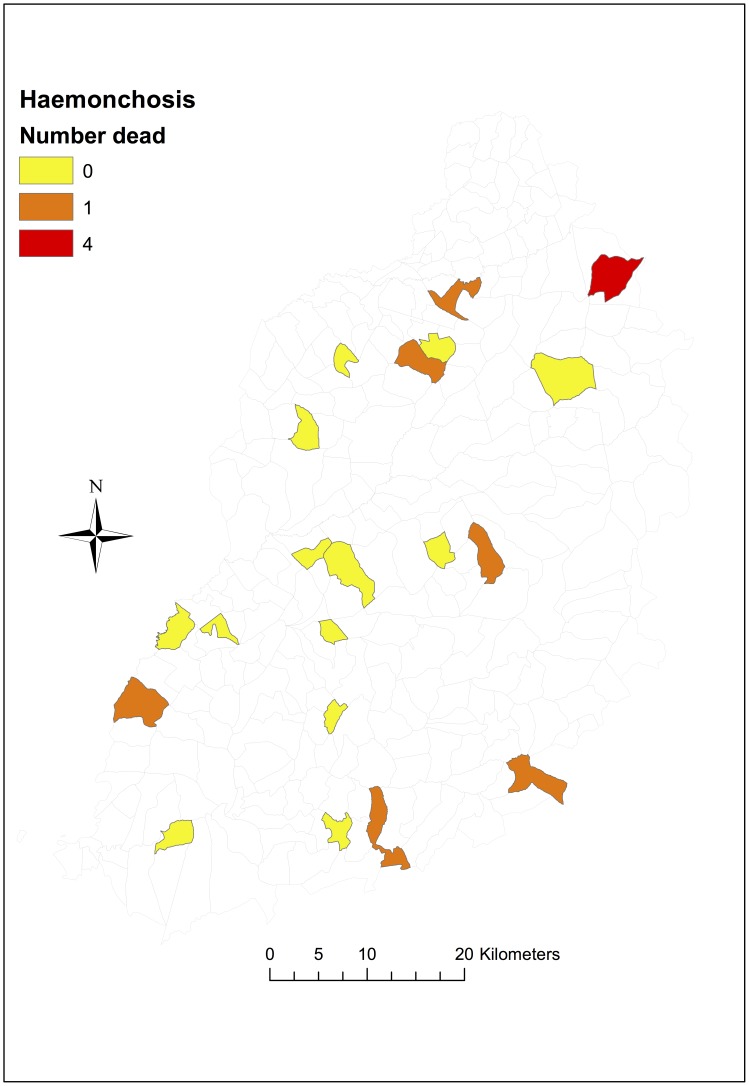
Map showing number of deaths attributable to haemonchosis by sub-location. In total, 10 of 88 deaths were attributed to haemonchosis.

The main aetiological causes of calf mortality among the indigenous zebu cattle were ECF and haemonchosis, accounting for 40% and 12% of all infectious disease deaths respectively. About 80% of deaths attributable to ECF occurred before calves were 6 months old, with only a few ECF deaths recorded in older calves, whereas deaths due to haemonchosis occurred in older calves, mostly greater than 6 months of age, see Figure 2. ECF deaths were observed across the study region, although Magombe East (in the south) and Bumala A recorded higher numbers of ECF deaths (6 and 4 respectively) compared to the other study sub-locations, see Figure 3. Deaths attributed to haemonchosis were observed in a number of the study sub-locations in low numbers (one death per sub-location), except in East Siboti sub-location located in the north where 4 deaths attributed to haemonchosis were recorded, Figure 4.

### Predictors for ECF Deaths

Putative non-infectious and infectious risk factors were initially run as univariable analyses to test their association with ECF deaths, see results in Table S1. Presence of a clinical episode and blood parameters such as packed cell volume, white blood cell count and total serum proteins were significantly associated with ECF-mortality. These variables were however not included in the multivariable analysis since they were considered a consequence rather than a cause of infection. High intensity (level 3– more than one infected cells per microscopy field) infection with *Theileria* spp. was associated with increased risk for ECF-mortality. This variable was left out in the multivariable analysis since these data had been used as part of the ECF-death case definition.

After controlling for other significant covariates in the model, co-infection with *Trypanosoma* spp. was estimated to increase the hazard for ECF death by 6 times (1.4, 25.8; 95% CI). In addition, the hazard for ECF death was increased by presence of strongyle eggs and this was burden dependent. An increase in strongyle eggs of 1000 was associated with a 1.5 times (1.4, 1.6; 95% CI) increase in the hazard for ECF mortality.

Sero-positivity to *T.parva* was identified to be associated with a protective effect against ECF-mortality. The risk hazard for ECF-mortality was reduced by 88% (78, 93; 95% CI) in animals that were sero-positive for *T.parva* compared to sero-negative animals.

Controlling for ticks within the farm in the rest of the herd was identified as the main husbandry practice associated with a protective effect against ECF-mortality. Farms that carried out tick control were associated with lowered hazard for ECF deaths by 54% (19, 75; 95% CI) compared to farms that did not control for ticks in the rest of the herd. The results of the minimum adequate model showing the predictors with significant association with ECF-mortality are provided in Table 2. Model diagnostics did not show evidence of violation of the proportional hazards assumption.

**Table 2:** Results of significant predictors of East Coast Fever deaths.

	HazardRatio	lowerCI	upperCI	*p-*value
Fixed effects				
Tick control	0.46	0.25	0.81	0.007
*T.parva* seroconversion	0.12	0.07	0.22	<0.001
*Trypanosoma* spp.	5.98	1.39	25.75	0.007
Strongyle eggs(per 1000 eggs)	1.48	1.37	1.61	<0.001
Group	Std Dev	Variance		
Sub-locationrandom effect	0.47	0.22		

### Predictors for Haemonchosis Deaths

The association between haemonchosis deaths and putative risk factors was initially tested through univariable analyses, see Table S2. While controlling for other covariates, results from the multivariable model revealed that calves from farms providing supplementary feeding had a 90% (48, 98; 95% CI) lower hazard for haemonchosis death compared to calves in farms that did not provide supplements. The main supplements provided to the calves were crop residues offered to the calves left at the homestead when adult cattle go grazing in the fields.

High worm burdens as measured by strongyle epg were associated with increased hazard for haemonchosis deaths with an estimated increase of 1.7 times (1.5, 2; 95% CI) in the hazard for every 1000 strongyle epg count increase. This finding indicates that the risk for haemonchosis death is burden-dependent. Since *H.placei* is a strongyle egg-producing helminth, the variable was omitted from the final model to test if the association of haemonchus deaths with the other covariates remained. The covariates remained significant in the absence of strongyle epg count in the model.

The results of the final model containing the significant predictors for haemonchosis deaths are provided in Table 3. Model diagnostics did not show evidence of violation of the proportional hazards assumption.

**Table 3:** Results of significant predictors of haemonchosis deaths.

	HazardRatio	lowerCI	upperCI	*p-*value
Fixed effects				
Supplements use	0.19	0.04	0.85	0.03
Strongyle eggs(per 1000 eggs)	1.67	1.43	1.94	<0.001
Group	Std Dev	Variance		
Sub-locationrandom effect	0.02	0.0004		

## Discussion

The findings presented here show that co-infections, which are common in areas endemic with diverse parasites, has important implications on host outcomes, in this study - calf survival. The study has investigated the risk factors for the two main causes of calf mortality in the study (ECF and haemonchosis) and tested the role co-infections play in determining the survival probabilities of zebu calves under one year.

East Coast fever, a disease caused by the protozoan parasite *Theileria parva* and transmitted by the tick *Rhipicephalus appendiculatus*, was identified as the main aetiological cause of death, accounting for 40% of all infectious disease calf mortality. Deaths due to ECF occurred mainly in young calves with up to 80% of deaths attributed to ECF occurring in calves below 6 months of age.

The risk of ECF death was itself significantly increased by high helminth burden (measured as strongyle epg) and by co-infection with *Trypanosoma* spp., evidence of co-infecting pathogens exacerbating the effect of infection with *T.parva*. An increase in strongyle egg per gram count of 1000 was associated with a 50% increase in hazard for ECF death, whereas co-infection with *Trypanosoma* spp. was associated with a 6 fold increase in the risk of dying from ECF. This is the first time such a result has been demonstrated and quantified in cattle, and underlines the importance of considering multiple infections in quantifying disease burden in conditions where polyparasitism is a rule rather than the exception.

The mechanisms by which *T.parva* and helminth infections interact to result in increased hazards for ECF deaths are unclear, and have not been described before. However, a similar co-infection profile involving *Plasmodium* spp., also a protozoan parasite, and helminth infections (including hookworms) has been the subject of many studies in humans, and in animal models. *Plasmodium* parasites are frequently occurring as co-infections with geohelminths, particularly hookworms with which they are co-distributed sharing extensive geographical overlaps in most of Africa [6]. Although the literature has conflicting results with reports of synergistic (increasing severity and incidence of malaria) and antagonistic (decreasing malaria cases) interactions [25]–[28] and reviewed by Nacher [29], most studies point to high helminth burden being associated with increased incidences and severity of malaria cases. More recently, a review by Adegnika and Kremsner [11] on the epidemiology of malaria and helminth interactions based on studies published in the last decade has concluded a general trend towards a worsening effect on the pathogenesis and incidence of malaria by hookworms and *Schistosoma mansoni*, and a protective effect by *Schistosoma hematobium* and *Ascaris lumbricoides*.

The interactions are thought to occur chiefly through immuno-regulation by helminth infections in two possible ways. First, the immune response becomes skewed to T-helper cell type 2 (Th2), required for fighting extracellular invaders, at the expense of T-helper cell type 1 (Th1) responses which are required for the control of microparasite infections including malaria parasitemia [30]. The second mechanism is through helminth induced immunomodulation that down-regulates both Th1 and Th2 responses, a strategy thought to be employed by helminths to avoid host immunity and possibly explaining why helminth infections even with known pathogenic species are often asymptomatic [31].

If similar mechanisms are at work with these study calves, a helminth skewed Th2 response and a dampened Th1 response would render a host co-infected with *T.parva* more susceptible to developing disease and affecting survival outcomes. Th1 responses are important for the generation for cytotoxic T lymphocytes (CTL), and if the helminth infections are skewing the response away from Th1 responses they may adversely affect the animal’s ability to generate CTL and thus its ability to control a *T.parva* infection. Here the risk for ECF death increases with helminth burden (measured by strongyle epg), which from larval cultures and identification of L3 show *Haemonchus placei* to be the main helminth producing strongyle eggs.

These results suggest co-infections with the hookworm *H.placei* may be playing a role in reducing the host’s ability to fight off *T.parva* infections. It is also possible that hookworms, which attach to the abomasal wall and suck whole blood, may be causing significant damage on their own weakening the calf more and increasing the risk of death with additional pathology from other co-infecting pathogens.

Trypanosomosis was not identified as a major cause of death in these cattle but the presence of trypanosome infections (mostly *T.vivax –* data not shown) increased the risk of death from ECF by up to 6 times. Like *T.parva*, infection with *Trypanosoma* spp. is known to lead to immuno-suppression. In addition, animals infected with trypanosomes have fever, lowered appetite, considerable weight loss, and anaemia. These effects coupled with immunosuppresion may lead to increased susceptibility and pathology in the host co-infected with *T.parva*.

Seropositivity to *T.parva* was associated with a protective effect against ECF-mortality. This result suggests that animals dying from ECF either die acutely before an antibody response that can be detected as a rising titre has occurred, or simply that the animals that do not mount an antibody response strong enough to be detected as sero-conversion are at a high risk of succumbing to an ECF infection. As naturally acquired antibodies are thought to play no role in resolving *T.parva* infections, seropositivity may reflect prior exposure rather than being a direct indicator of immune status. If ECF death is acute it would be interesting to know why some animals survive first exposure (evidence by seropositivity) and others die on first infection. The intensity of *Theileria* spp. infection, specifically level 3 infection - multiple infected cells in multiple microscopy fields, was identified to be associated with a high risk for mortality. The risk for death, it appears, is related to the intensity of infection which may be the result of a high dose of infection or an indication of a host unable to control the within host multiplication of the infecting pathogen.

Results of the analysis of risk factors associated with ECF deaths revealed controlling for ticks in a farm was associated with a protective effect. The risk of ECF-death in farms carrying out tick control was 80% lower than in farms not controlling for ticks. Tick control was not done on the study calves and the observed protective effect is a benefit associated with control in the rest of the herd.

Infections with *Haemonchus placei* were themselves identified as the second most important aetiological cause of mortality in zebu calves. A high burden of strongyle eggs was identified as a significant predictor for deaths due to haemonchosis, pointing to their impact being burden-dependent. *H.placei* accounted for more that 80% of all larvae hatched following incubation of the strongyle eggs.

Farms that reported providing supplements (mainly crop residue) to the animals had significantly lower hazards for haemonchosis deaths than those that did not provide supplements. Calves in such farms are fed mainly while within the homesteads, and as a result will visit grazing pastures less frequently or take longer before starting to access communal grazing fields. These factors reduce the exposure to helminths, and may explain the association between supplement feeding and risk for deaths due to haemonchosis. It is also possible that animals receiving supplementation have improved nutrition reducing the effects of helminthosis.

The findings of this study suggest reduction in calf mortality would be attained through improved husbandry practices to reduce levels of exposure to pathogens to calves. Reducing the burden of livestock diseases, including exacerbated burden associated with pathogen-pathogen interactions during co-infections, is crucial if livestock are to be a viable pathway out of poverty. This is especially important in protecting livestock assets belonging to people living in poverty against mortality, and reducing losses in production associated with these diseases [32], [33]. The results suggest that integrated tick, trypanosomes and worm-control programs would likely have large benefits in not just reducing mortality due to individual diseases, but also excess co-infection exacerbated mortality. Such integrated control programs have been suggested for example in the control of anaemia-related burden of malaria in humans, which is worsened by high hookworm burden [6].

## Supporting Information

Table S1 **Results of survival analysis univariable screening for infectious and non-infectious predictors of ECF mortality (33 cases).** The table contains all risk factors with a *p-*value ≤0.2 and that were offered to the multivariable analysis.
(DOCX)

Table S2 **Results of survival analysis univariable screening for infectious and non-infectious predictors of deaths attributable to haemonchosis (10 cases).** The table contains all risk factors with a *p-*value ≤0.2 and that were offered to the multivariable analysis.
(DOCX)
