# Supplementary material for: Parasite Co-Infections and Their Impact on Survival of Indigenous Cattle
Source: PLoS One. 2014 Feb 20;9(2):e76324. doi: 10.1371/journal.pone.0076324 (PMC3930515; doi:10.1371/journal.pone.0076324)
Supplement: Table S1 — Results of survival analysis univariable screening for infectious and non-infectious predictors of ECF mortality (33 cases). The table contains all risk factors with a p-value ≤0.2 and that were offered to the multivariable analysis. (DOCX) [file pone.0076324.s001.docx]

Supplementary Table S1: Results of survival analysis univariable screening for infectious and non-infectious predictors of ECF mortality (33 cases). The table contains all risk factors with a *p-*value ≤ 0.2 and that were offered to the multivariable analysis.

| **Variable** | **exp(coef)** | **se(coef)** | ***p-*value** |
| --- | --- | --- | --- |
| Education-Primary school | 0.46 | 0.41 | 0.057 |
| Education-Secondary school | 0.41 | 0.53 | 0.090 |
| log(Tropical livestock units) | 1.40 | 0.24 | 0.165 |
| Farmer's age | 1.03 | 0.01 | 0.041 |
| Housing calves – yes | 2.17 | 0.60 | 0.198 |
| Suckling-yes | 0.17 | 0.78 | 0.025 |
| Supplements use – yes | 0.50 | 0.38 | 0.070 |
| Housing stall-shed | 0.57 | 0.35 | 0.106 |
| Worm control – yes | 0.37 | 0.53 | 0.062 |
| Antibiotics use – yes | 0.31 | 0.45 | 0.010 |
| Tick control – yes | 0.19 | 0.37 | 0.001 |
| Mean NDVI | 0.00 | 3.41 | 0.019 |
| Heart girth size – dam | 0.96 | 0.03 | 0.136 |
| Body condition score – dam | 0.66 | 0.20 | 0.043 |
| Health of dam – sick | 10.99 | 1.03 | 0.020 |
| *B.bigemina* antibodies – dam | 1.01 | 0.01 | 0.040 |
| *T.parva* antibodies – dam | 1.02 | 0.01 | 0.019 |
| Clinical episode | 13.29 | 0.37 | 0.001 |
| Total serum proteins | 0.35 | 0.20 | 0.001 |
| White blood cell count | 0.76 | 0.07 | 0.001 |
| Packed cell volume | 0.81 | 0.03 | 0.001 |
| **Pathogens** |  |  |  |
| *T.parva* seropositivity | 0.15 | 0.53 | 0.001 |
| *T.mutans* seropositivity | 0.22 | 0.48 | 0.002 |
| *A.marginale* seropositivity | 0.24 | 0.75 | 0.054 |
| *Anaplasma* spp. | 4.19 | 1.02 | 0.160 |
| *Trypanosoma* spp. | 7.82 | 0.74 | 0.005 |
| *Trypanosoma vivax* | 6.78 | 1.03 | 0.063 |
| *Calicophoron* spp. | 2.00 | 0.51 | 0.171 |
| *Fasciola* spp. | 4.32 | 1.06 | 0.167 |
| *Haemonchus placei* | 2.33 | 0.44 | 0.055 |
| Strongyle eggs | 3.23 | 0.50 | 0.020 |
| *Trichophyton* spp. | 4.14 | 1.03 | 0.167 |
| *Theileria* spp level 1 | 0.61 | 0.45 | 0.262 |
| *Theileria* spp level 2 | 2.18 | 0.77 | 0.312 |
| *Theileria* spp level 3 | 20.19 | 0.75 | 0.000 |
| Strongyle eggs/1000 | 1.33 | 0.08 | 0.001 |
