# Supplementary material for: Parasite Co-Infections and Their Impact on Survival of Indigenous Cattle
Source: PLoS One. 2014 Feb 20;9(2):e76324. doi: 10.1371/journal.pone.0076324 (PMC3930515; doi:10.1371/journal.pone.0076324)
Supplement: Table S2 — Results of survival analysis univariable screening for infectious and non-infectious predictors of deaths attributable to haemonchosis (10 cases). The table contains all risk factors with a p-value ≤0.2 and that were offered to the multivariable analysis. (DOCX) [file pone.0076324.s002.docx]

Supplementary Table S2: Results of survival analysis univariable screening for infectious and non-infectious predictors of deaths attributable to haemonchosis (10 cases). The table contains all risk factors with a *p-*value ≤ 0.2 and that were offered to the multivariable analysis.

| **Variable** | exp(coef) | se(coef) | *p-*value |
| --- | --- | --- | --- |
| Suckling-yes | 0.19 | 0.81 | 0.043 |
| Watering at homestead | 0.36 | 0.69 | 0.143 |
| Distance to water < 1km | 0.45 | 0.65 | 0.222 |
| Supplements use – yes | 0.33 | 0.65 | 0.084 |
| Mean NDVI | 0.00 | 6.41 | 0.174 |
| Elevation | 1.01 | 0.01 | 0.017 |
| Heart girth size – dam | 1.02 | 0.05 | 0.774 |
| *B.bigemina antibodies – dam* | 1.02 | 0.01 | 0.145 |
| *T.parva antibodies – dam* | 1.02 | 0.01 | 0.078 |
| Moderate introgression | 2.96 | 0.71 | 0.124 |
| Clinical episode | 42.75 | 0.70 | 0.001 |
| Total serum proteins | 0.02 | 0.63 | 0.001 |
| White blood cell count | 0.61 | 0.12 | 0.001 |
| Packed cell volume | 0.40 | 0.22 | 0.001 |
| **Pathogens** |  |  |  |
| *T.parva* seropositivity | 0.25 | 0.65 | 0.033 |
| *Nematodirus spp.* | 0.51 | 1.09 | 0.016 |
| *Oesophagostomum radratium* | 4.23 | 0.65 | 0.027 |
| *Calicophoron spp./1000* | 1.00 | 0.79 | 0.036 |
| Strongyle eggs/1000 | 1.63 | 0.07 | 0.001 |
